# The role of exosomal transport of viral agents in persistent HIV pathogenesis

**DOI:** 10.1186/s12977-018-0462-x

**Published:** 2018-12-22

**Authors:** Benjamin J. Patters, Santosh Kumar

**Affiliations:** Pharmaceutical Sciences, University of Tennessee Health Science Center, Memphis, TN USA

**Keywords:** Exosome, HIV, HAND, Tetraspanin, ESCRT

## Abstract

Human immunodeficiency virus (HIV) infection, despite great advances in antiretroviral therapy, remains a lifelong affliction. Though current treatment regimens can effectively suppress viral load to undetectable levels and preserve healthy immune function, they cannot fully alleviate all symptoms caused by the presence of the virus, such as HIV-associated neurocognitive disorders. Exosomes are small vesicles that transport cellular proteins, RNA, and small molecules between cells as a mechanism of intercellular communication. Recent research has shown that HIV proteins and RNA can be packaged into exosomes and transported between cells, to pathogenic effect. This review summarizes the current knowledge on the diverse mechanisms involved in the sorting of viral elements into exosomes and the damage those exosomal agents can inflict. In addition, potential therapeutic options to counteract exosome-mediated HIV pathogenesis are reviewed and considered.

## Background

Since the discovery of human immunodeficiency virus (HIV) as the causative agent in the AIDS epidemic of the 1980s and 90s, advancements in antiretroviral therapy (ART) have vastly improved life expectancy and quality of life for HIV-infected individuals. However, being a lifelong infection, HIV continues to present a significant threat to public health. The Centers for Disease Control determined that there were approximately 974,000 people in the United States living with diagnosed HIV infection in 2015, with approximately 40,000 new cases being diagnosed annually since 2011 [1]. This large infected population faces a number of additional health concerns such as ART-induced toxicity, toxic drug–drug interactions, viral resurgence due to poor ART adherence, neurocognitive dysfunctions, and the rising occurrence of drug resistance in some HIV strains, which all present a growing healthcare challenge [2].

The current standard for ART is treatment with a cocktail of drugs that each inhibit a separate stage of the viral life cycle: entry and membrane fusion, reverse transcription of the viral genome, integration of viral DNA into the host genome, and assembly and maturation of new virions [3, 4]. Combination therapy with multiple types of antiretroviral drugs helps to prevent drug resistance mutations by providing several simultaneous barriers to reproduction [4].

Despite viral suppression and restoration of immune function with ART, the virus often establishes infection within the central nervous system, transporting across the otherwise impermeable blood–brain barrier (BBB) and causing neuroinflammation, astrocytosis, and neurodegeneration, which can lead to a spectrum of neurological conditions collectively referred to as HIV-associated neurocognitive disorders (HAND) [5, 6]. HIV patients on regular ART have a reduced risk of severe neurocognitive impairment compared to patients who do not have access to ART, but still greater incidence of neurological symptoms compared to the uninfected population [7, 8]. Furthermore, ART has not been able to reduce the frequency of neurocognitive disorders in HIV patients, though it has significantly reduced their overall severity [9]. ART penetration into the central nervous system is generally correlated with better cognitive performance [10], however there is some controversy on that point, as some studies have failed to reproduce that relationship [11]. There is also disagreement on whether neurocognitive impairment in HIV patients receiving ART is necessarily a product of viral replication in the central nervous system, or a product of sustained neuroinflammation or another neurotoxic byproduct of HIV infection [12].

Considering this, the inability of current ART regimens to eliminate the symptoms of HIV-induced neurodegeneration, despite healthy resting CD4 counts and undetectable viral loads in HIV patients, could be due not to insufficient ART penetration and uncontrolled viral replication, but rather to another mechanism of viral pathogenesis that is not yet completely understood. One such mechanism, the subject of only recent investigation, is the potential role that exosomes may play in contributing to replication, secretion, and/or toxicity during HIV infection.

Exosomes are small membrane-bound vesicles, approximately 100 nm in diameter, that are secreted from and taken up by almost every type of cell [13]. They are born out of the endocytic pathway: clathrin-mediated invagination of the plasma membrane causes formation of an early endosome, which in turn buds inwards and forms multiple intraluminal vesicles [14, 15]. This multivesicular body (MVB) then, instead of fusing with a lysosome and having its contents digested, returns to fuse with the plasma membrane and release those vesicles into the extracellular space as exosomes [16]. Originally believed to be a mechanism of waste disposal for the elimination of proteins from the plasma membrane [17, 18], it has since been discovered that exosomes also serve as mediators of intercellular communication by transporting proteins, RNA, and small molecules [19]. The packaging of exosomes with these biological cargo is likely not only mediated by undirected capture of membrane-bound and cytosolic proteins and RNA, but also a specific and directed process of cargo sorting. The endosomal sorting complex required for transport (ESCRT), an ancient and evolutionarily conserved complex of proteins that mediates scission of lipid membranes and is involved in MVB formation and exosome release [20], has been shown to interact with both protein and RNA trafficking machinery to facilitate vesicle packaging [21]. There is also evidence of ESCRT-independent directed packaging of some exosomal cargo, mediated by membrane tetraspanins such as CD63 and CD81, two reliable exosomal marker proteins [22].

Once released, exosomes diffuse through body fluids such as blood, saliva, lymph, and spinal fluid until they come into contact with recipient cells which then take them up by multiple mechanisms [23, 24]. The effects that the delivery of exosomal contents have on the recipient cells are myriad, and can be both beneficial and deleterious [25–27]. Exosome contents are subject to change in response to various stimuli, selectively packaging different proteins and RNA in response to conditions such as hypoxia, exposure to xenobiotic compounds such as ethanol, or signaling molecules like cytokines [28, 29]. Investigation of the contents of exosomes under various conditions has enormous diagnostic potential, allowing for early detection of various cancers and many other progressive diseases [30, 31]. In the case of HIV infection, there is evidence that the virus alters exosomal content both directly and indirectly, and utilizes the exosome secretion pathway to enhance its own reproduction and pathogenesis. In this review, the mechanisms by which HIV may hijack exosome production machinery will be discussed. The known downstream effects of exosomal transport of HIV elements will also be reviewed, and potential therapeutic options will be considered.

## Viral agents are secreted from infected cells via exosomes

While the most obvious mechanism of HIV pathogenesis is the direct killing of infected T-cells by the replicating virus, it also has other methods of causing the associated inflammation, immune depletion, and neurodegeneration. It was discovered in the early 1990s that HIV-infected cells also secrete viral proteins directly into the extracellular space. For example, in 1990, Ensoli et al. [32] described extracellular secretion of the HIV Tat protein from CD4+ T-cells, which enhanced the growth of Kaposi’s sarcoma-like lesions in mice. In 1994, Levy et al. [33] reported that the HIV structural protein Vpr was present in both the serum and cerebrospinal fluid of AIDS patients, and 2 years later, Fujii et al. [34] reported a similar finding of soluble Nef protein in patient sera.

In general, the secretion of these proteins is proportional to viral load [35], but is not necessarily eliminated by ART [36], presenting a continuing health challenge for patients living with HIV. These secreted proteins enhance viral pathogenesis by multiple mechanisms, which will be discussed subsequently in more detail.

However, it only recently came to light that the secretion of free viral proteins into the extracellular space is not the only mechanism of HIV-mediated intercellular contact. In fact, recent research has shown that a number of different HIV proteins have been found packaged within exosomes. Lenassi et al. [37], for instance demonstrated that the HIV protein Nef was packaged into exosomes in both infected and transformed T-cells, and Pužar Dominkuš et al. [38] reported similar observations in astrocytes. This has been reported to be an evolutionarily conserved phenomenon, as it has also been found to occur in simian immunodeficiency virus, a closely related retrovirus that infects numerous primate species [39]. HIV Tat has also been found within exosomes secreted from transfected astrocytes [40], and the same lab also observed the viral capsid protein p24 in vesicles collected from Jurkat T-cells [41]. Additionally, the viral envelope protein gp120 was discovered by Arakelyan et al. [42] in extracellular vesicles within stock viral preparations. Perhaps the most extensive array of vesicular HIV proteins comes from a recent report by Anyanwu et al. [43], who detected a large variety of viral proteins, including Nef, Tat, Vpr, and uncleaved Gag and Pol peptides, in extracellular vesicles collected from the urine of HIV patients. It is worth noting, however, that the vesicles were not positively identified as exosomes, and that these results have not yet been reproduced.

Not only viral proteins, but also RNA, can find its way into exosomes. In 2013, Narayanan et al. [44] reported that exosomes from HIV patient sera and primary T-cells infected with HIV ex vivo contained trans-activation response element (TAR) RNA. TAR is part of a short untranslated region of the HIV genome that serves as a binding site for Tat, enhancing viral transcription. It also encodes viral microRNAs that protect infected cells against apoptosis [45, 46]. The same laboratory later confirmed that TAR was also packaged into exosomes from infected monocytes and microglia [47], and an independent study in Slovenia later confirmed the presence of TAR in exosomes from the plasma of aviremic HIV-positive subjects [48]. Interestingly, while Narayanan et al. reported little to no exosomal packaging of unspliced HIV RNA into exosomes, a follow-up study from the same laboratory revealed the presence of both TAR and full-length genomic RNA in exosomes from the plasma of HIV patients, though the TAR RNA was in much greater abundance, implying a specific sorting mechanism that differs from normal mechanisms that package viral RNA into virions [49]. This supported the findings from another study by Columba Cabezas and Federico [50] which found that the whole unspliced viral genome was readily packaged into exosomes in monocytes and transfected HEK293T cells in vitro.

The vesicular export of viral components is not unique to HIV. Epstein-Barr virus, hepatitis B virus, and Rift Valley fever virus, amongst others, also package viral proteins into exosomes and other extracellular vesicles [51, 52]. Viral mRNA has been reported as well in extracellular vesicles derived from cells infected with human pegivirus, hepatitis C virus (HCV), and the fellow retrovirus human T-lymphotropic virus [53]. What may distinguish HIV from these other viruses, at least for the time being, is the amount of research that has been done on the potential mechanisms by which the packaging of its components into exosomes.

In 2003, Gould et al. proposed a controversial hypothesis concerning HIV reproduction, dubbed the “Trojan Exosome Hypothesis”, in reference to the earlier Trojan Horse Hypothesis which described a possible mechanism of HIV neuroinvasion [54, 55]. The hypothesis stated that, in addition to the canonical method of reproduction via direct budding from the plasma membrane, HIV has also evolved to interact with the exosome formation and packaging pathways, budding into the early endosome and being released as the MVB fuses with the cell membrane [55]. The hypothesis went so far as to suggest that the exosomal pathway may have been the origin of the virus itself; in other words, that HIV itself may be an exosome that acquired virus-like replicative capability and became a distinct particle [56]. This controversial hypothesis initially had some strong support: viral particles collected from HIV-infected monocyte-derived macrophages (MDM) bear a number of host proteins in common with exosomes, including the major histocompatibility complex class II and the exosomal marker proteins CD63 and CD81 [57, 58]. Furthermore, a study by Sherer et al. [59] appeared to show that the viral polyproteins Gag and Env accumulate at the membrane of the late endosome and MVB and mediate assembly at those sites.

The Trojan Exosome Hypothesis quickly encountered resistance [60, 61]. In 2007, two independent laboratories published reports that HIV particles localized and budded at invaginations in the plasma membrane of MDMs that could previously have been mistaken for intracellular endosomes, as they were rich in the same membrane-bound marker proteins, including CD63 and CD81 [62, 63]. Later, Grigorov et al. [64] reported similar findings of HIV polyprotein localization to the plasma membrane in association with CD81 in infected T-cells. These reports provided a potential explanation for the presence of the exosomal markers in the membrane of viral particles, as well as an alternative interpretation of the data presented by Sherer et al. Later, Park and He [41] and Coren et al. [65] also presented findings that T-cells cells secrete exosomes and viral particles by separate processes, as exosomes package cellular beta-actin protein, whereas virions do not.

## Mechanisms of exosomal packaging of HIV elements

While the Trojan Exosome Hypothesis remains a subject of discussion and debate, research by the original proponents of the hypothesis was foundational in uncovering some of the mechanistic elements behind the secretion of viral proteins, particularly Gag, into extracellular vesicles, if not specifically exosomes. Fang et al. [66] demonstrated that higher-order oligomerization, i.e. oligomerization of multiple Gag peptides, at the plasma membrane contributes to vesicular export of the polypeptide by an apparently sequence-independent process. There has been very little research regarding how higher-order oligomerization affects protein loading into the MVB or canonical exosomes, however, so the relevance and implications of this phenomenon with regard to exosomal transport of host or viral proteins are not yet understood.

The Gag and Env polyproteins, as reported by Jolly et al. and others, localize to regions of the plasma membrane that are enriched in tetraspanins such as CD81 and CD63, prior to assembly and budding [64, 67]. Jager et al. have shown that Gag can interact directly with CD81 and CD9, and Booth et al. demonstrated that association between Gag and CD81 at the plasma membrane led to secretion of Gag within extracellular vesicles [56, 68]. It should be noted though, that while Booth et al. [56] refer to these vesicles as “exosomes”, they are not derived from the MVB. Rather they bud directly from the plasma membrane, much like a virus. These vesicles are referred to in current parlance as ‘ectosomes’, and while they share much in common with exosomes with regard to their protein profile and process of membrane budding and scission, they feature less specific packaging with regard to their contents [69]. Nevertheless, the regions of the plasma membrane that are enriched with these tetraspanins, referred to as tetraspanin-enriched microdomains (TEMs), in addition to being sites of HIV aggregation and budding [70], are also sites for membrane invagination and early endosome formation, the first step in exosome biogenesis. This is evidenced by their enrichment in the intraluminal vesicles of the MVB relative to the endosomal and plasma membranes [71]. In 2013, Perez-Hernandez et al. demonstrated that direct interactions with TEM proteins, particularly CD81, leads to specific packaging of proteins into exosomes [22, 72]. Thus, while it has not yet been specifically demonstrated, it is likely that the localization of Gag at TEMs, primarily for the purpose of assembly and budding at the plasma membrane, also results in tetraspanin-mediated packaging of Gag into endosomes, MVBs, and ultimately exosomes. Significantly, given the capability to bind to other viral elements such as Env and genomic RNA in order to facilitate virion assembly [73], this may be a mechanism by which those elements are sorted into exosomes as well, though this hypothesis has yet to be demonstrated.

A similar phenomenon may also occur with lipid rafts. Lipid rafts are regions of the membrane that are rich in cholesterol and unsaturated fats. Much like TEMs, lipid rafts serve as sites of viral assembly and budding [74, 75]. There are also some reports that lipid rafts may be preferentially endocytosed and exported via exosomes [76, 77]. There is also evidence, as reported by Hogue et al. and others from the same group, that Gag can induce overlap of lipid rafts and TEMs to enhance viral assembly and budding, though the implications for exosome formation and packaging are not clear at this time [75, 78].

Another mechanism for viral protein packaging is via the ESCRT machinery. Some elements of the ESCRT complex, which plays a part in regulating the creation, cargo sorting, and release of vesicles, are known to interact with HIV and contribute to viral budding [20, 21]. The HIV Gag peptide has two short amino acid domains that are known to bind elements of the ESCRT complex, both within the C-terminal p6 protein. The first is the primary late assembly domain, with the sequence PTAP (Pro-Thr-Ala-Pro), which interacts with Tsg101, a subunit of ESCRT-I. The second is the auxiliary late assembly domain, which has the sequence LYPXnL (Leu-Tyr-Pro-Xaan-Leu) and interacts with Alix, an ESCRT-III-associated mediator of vesicle creation [79, 80]. As the ESCRT complex is closely involved in the formation and loading of exosomes, Alix and Tsg101 are also often associated with them, to the degree that they are frequently used as secondary exosomal markers [81]. Given the known associations between these elements of the ESCRT complex and Gag, as well as their role in the sorting of proteins to exosomes, it is likely that they may specifically load Gag and potentially other viral proteins into exosomes as well, though this has not yet been demonstrated.

Little has been reported concerning the mechanisms by which HIV Tat protein may be incorporated into exosomes, though Mele et al. [82] speculated on this subject in their recent review of the mechanisms of Tat secretion. One method they suggest is by association of Tat with phosphatidylinositol-4,5-bisphosphate, a phospholipid that facilitates direct secretion of Tat across the plasma membrane via binding and pore formation. Phosphatidylinositol-4,5-bisphosphate is present within the membrane of the early endosome and MVB, and could allow Tat to translocate from the cytosol into the intraluminal vesicles of the MVB, much as it has been demonstrated to do at the plasma membrane [76, 82]. Mele et al. also suggest another potential mechanism: that Tat may bind to transcribed viral TAR RNA, or to host miRNAs, tethering itself to them as they are packaged into exosomes by RNA binding proteins. Sutaria et al. [83] recently engineered an HIV Tat/Lamp2a fusion protein that, when paired with a pre-miRNA target containing the sequence for the TAR loop, dramatically enhances loading of the RNA into extracellular vesicles positive for Tsg101. However, this loading process was mediated by the Lamp2a peptide, which is membrane-bound and packaged into the MVB under physiological conditions, and does not demonstrate that normal Tat-TAR binding is sufficient to facilitate loading either viral element into exosomes.

As for loading of TAR RNA itself, as yet not much is known. In 2013, Narayanan et al. reported the presence of TAR in exosomes collected both in vitro and ex vivo. In their study, they also found Dicer and Drosha, two principle proteins of the RNA interference machinery, of which Dicer is known to bind and process TAR [44, 84]. A cancer study by Melo et al. [85] found that Dicer could be loaded into exosomes via interaction with CD43, a membrane anchor protein. As such, packaging of TAR could be facilitated by CD43-mediated transport of TAR-bound Dicer, similar to the mechanism demonstrated by Sutaria et al. with their Lamp2a fusion protein. Janas et al. [86] have also proposed a general mechanism of exosomal RNA loading mediated by direct interaction between sequence motifs of RNA molecules with the lipid raft-like domains of the MVB outer membrane. While the canonical TAR sequence does not contain any of the specific motifs reported by Janas, and there is no supporting evidence that this phenomenon occurs in the case of TAR packaging, it remains a potential avenue of viral RNA packaging worth further investigation.

Perhaps the most research into the packaging of HIV elements into exosomes has concerned the viral protein Nef. Nef is a nonstructural accessory protein that facilitates viral infection and pathogenesis by interacting with a multitude of cellular pathways, including endocytosis and intracellular trafficking [87]. Its packaging into extracellular vesicles has been well established in T-cells and transfected HEK293 cells [88, 89], and it has been found to be secreted via both exosomes and other microvesicles, depending on the cell of origin [37]. One mechanism that could be at play is association with lipid rafts. It has been well established that Nef binds and anchors to lipid raft domains of the plasma membrane, enabling some but not all of its pathogenic effects [90, 91]. This association, along with the previously mentioned enrichment of lipid rafts in MVBs [77] may lead to piggybacking of the tethered Nef protein into exosomes [92]. Work by Ali et al. [93] has shown that Nef has multiple sequence motifs, mostly within the first 70 amino acids from the N-terminus, that are necessary for its exosomal secretion. A follow-up study by the same group identified a five amino acid sequence, dubbed the secretion modification region, that facilitated exosomal packaging though binding with mortalin, a heat shock protein with a known ability to contribute to vesicular protein sorting [94, 95]. Nef is also able to greatly enhance exosome and microvesicle secretion from T-cells and transfected HeLa cells by a mechanism that has not yet been fully identified [37, 96]. The diversity of HIV elements that can be sorted into exosomes, of the mechanisms involved in that process, and of the types of potential sources of those exosomes, creates a complex picture of the role of those vesicles in HIV pathogenesis (Table 1). Viral elements may be loaded to differing extents or by distinct processes in various infected cell types, possibly contributing to the multiple pathogenic consequences of HIV-exosomal uptake.

**Table 1:** HIV elements packaged within exosomes and their effects

HIV element	Source	Effect	References
Nef protein	Plasma	Enhanced amyloid beta secretion	Khan et al. [117]
		Activation-induced T-cell death	Raymond et al. [104]
	T-cells	Activation-induced T-cell death	Lenassi et al. [37]
			Konadu et al. [105]
		Viral reactivation from latency	Arenaccio et al. [98]
			Arenaccio et al. [99]
		n/a	Muratori et al. [96]
	MDMs	Inflammatory cytokine production	Arenaccio et al. [99]
	Microglia	Reduced BBB integrity	Raymond et al. [107]
	Astrocytes	n/a	Pužar Dominkuš et al. [38]
	Transfected HeLa cells	Activation-induced T-cell death	Lenassi et al. [37]
	Transfected HEK cells	Activation-induced T-cell death	Raymond et al. [104]
		n/a	Campbell et al. [88]
Tat protein	T-cells	Activation of viral promoter	Rahimian and He [40]
	Astrocytes	Activation of viral promoter	Ibid.
		Neurite shortening and neurotoxicity	Ibid.
	Transfected HEK cells	Activation of viral promoter	Ibid.
Gag protein	MDMs	Enhanced infection	Kadiu et al. [146]
	T-cells	n/a	Narayanan et al. [44]
Env protein	T-cells	n/a	Ibid.
	n/a	Enhanced infection	Arakelyan et al. [42]
TAR RNA	Plasma	Enhanced infection	Narayanan et al. [44]
	T-cells	Protection against extrinsic apoptosis	Ibid.
		Inflammatory cytokine production	Sampey et al. [100]
		n/a	Barclay et al. [47]
	MDMs	Inflammatory cytokine production	Sampey et al. [100]
	Microglia	n/a	Barclay et al. [47]
Viral miRNAs	MDMs	Inflammatory cytokine production	Bernard et al. [101]

## Consequences of exosomal delivery of HIV elements

Packaging of viral contents into exosomes allows for their transport to other cells, expanding the reach of the virus’s various destructive effects on the host. One consequence of this exosomal delivery is the reactivation of viral replication from latent cells. Tang et al. artificially loaded exosomes from transfected HEK293T cells with HIV Tat protein, and treated the exosomes to primary HIV-infected resting CD4+ T-cells. The exosomal Tat reactivated HIV replication in those cells through binding at the 5′ long terminal repeat portion of the genome, the site of the TAR sequence [105]. Kadiu et al. demonstrated that exosomes and microvesicles from infected MDM, which were positive for Gag-derived peptides, enhanced the infectivity of the virus during co-treatment to MDM, by an unclear mechanism [106].

A series of papers from an Italian laboratory recently explored how exosomes from infected cells can induce viral replication. They found that infected T-cells released exosomes containing active ADAM17, a cellular protease which induced activation and replication of HIV in recipient T-cells. This packaging of ADAM17 only occurred if the exosomes contained HIV Nef, as cells infected with mutant strains that had Nef which was incapable of binding to membranes failed to package either protein or to activate downstream viral replication [97]. In a follow-up study, they found that the exosomal ADAM17 induced this activation via cleavage of pro-TNF-α to the mature form of the proinflammatory cytokine [98]. Further research demonstrated that this phenomenon also occurs with exosomes derived from HIV-infected MDMs as well [99].

A likely secondary consequence of the mechanism proposed by Arenaccio et al. is inflammation resulting from the activation of both infected and uninfected resting T-cells via TNF-α. But that is not the only means by which exosomes from HIV-infected cells may provoke inflammation. Sampey et al. [100] showed that exosomes bearing TAR RNA induce secretion of pro-inflammatory cytokines, specifically TNF-ß and IL-6, from MDM via binding to the toll-like receptor 3 protein and subsequent activation of the NF-kB pathway. Bernard et al. also showed the proinflammatory potential of exosomal packaging of viral RNA. They demonstrated that primary human alveolar macrophages secreted exosomes containing viral microRNAs (dubbed vmiR88 and vmiR99) that stimulated activation of recipient macrophages which then released TNF-a. This induction was likely mediated by binding of the guanine and uracil-rich single-stranded vmiRNAs to toll-like receptor 8 and stimulation of the NF-kB pathway [101]. It is also of note that NF-kB directly enhances transcription of the HIV genome through binding to its 5′ LTR region [102], which indicates that viral mechanisms of inducing inflammation are likely to also promote further HIV replication [103].

Another avenue of HIV pathogenesis is immunodeficiency through the depletion of uninfected bystander T-cells. In the report by Lenassi et al. [37] mentioned previously, exosomal Nef protein from infected T-cells caused activation-induced apoptosis in uninfected recipient cells. Raymond et al. [104] and Konadu et al. [105] have both published similar observations of vesicular Nef inducing apoptosis in T-cells, though notably not in MDMs. A study by Muratori et al. [96] showed that exosomal Nef induced expression and secretion of the Fas ligand, a mediator of activation-induced T cell death. This is an established mechanism of Nef-mediated immunotoxicity [106], however the fact that it occurs via exosomal transfer of Nef even in patients on ART is of great clinical significance.

Exosomal Nef also negatively affects endothelial cells. In a recent study, Raymond et al. presented data demonstrating that exosomes from Nef-transfected microglia could disrupt the integrity of an in vitro model of the BBB. The authors further showed that this disruption was due at least in part to a Nef-induced downregulation of zona occludin-1, also referred to as tight junction protein 1, which they hypothesized weakened the tight junctions between endothelial cells [107]. The effects of secreted Nef, exosomal and otherwise, on tight junction integrity has led to speculation that exosomes containing Nef may contribute to endothelial disruption in the gut as well, though there is not much evidentiary support for that hypothesis as of yet [108]. It is also of note that other HIV proteins such as Tat, Vpr, and gp120 also weaken the BBB by several mechanisms, such as downregulation and/or oxidative stress-induced phosphorylative dysregulation of tight junction proteins [109]. While it has not yet been explicitly demonstrated, it is likely that exosomal transport of these proteins contributes to BBB leakiness and viral neuro-invasion in vivo.

Perhaps the most clinically significant consequence of viral exosome production is its potential impact on neuroinflammation, neurodegeneration, and HAND. It has been well established that HIV proteins are primary mediators of neurological dysfunction. For example, gp120, the Env-derived surface glycoprotein, induces apoptosis in neurons through binding with the *N*-methyl-d-aspartate receptor, triggering excitotoxic cell death [110, 111]. Tat can also cause excitotoxicity through a similar mechanism, and also induces oxidative stress in neurons via downstream induction of spermine oxidase [112]. Ferrucci et al. [113] have written extensively on the multiple deleterious effects of extracellular Vpr protein in the central nervous system, including disruption in action potential conduction, mitochondrial disruption, induction of oxidative stress, and triggering p53-mediated apoptosis in astrocytes. Systems of intercellular communication such as exosome transport, however, have only recently begun to be studied in the context of HIV neuropathogenesis [114, 115].

What little research has been published on this subject so far has shown that vesicular transport of viral proteins within the central nervous system is likely to exacerbate viral neurotoxicity, much as free protein does. Rahimian and He showed that exosomes from Tat-transfected T-cells and astrocytes induced neurite shortening and cell death in recipient neurons, a phenomenon also observed when neurons are treated with free recombinant Tat [40, 116]. A study by Khan et al. [117] presented evidence that exosomes containing Nef protein and mRNA could induce production and secretion of amyloid beta protein from neuroblastoma cells in vitro. Excessive amyloid beta production is known to be neurotoxic, and is a hallmark of Alzheimer’s disease as well as an indicator of poor prognosis in cases of age-related HAND [118]. Interestingly, András et al. [119] recently demonstrated that HIV exposure could induce secretion of amyloid beta in exosomes from BBB endothelial cells, which implies that vesicular packaging of host proteins could be yet another mechanism of HIV neuropathogenesis.

**Fig. 1:**
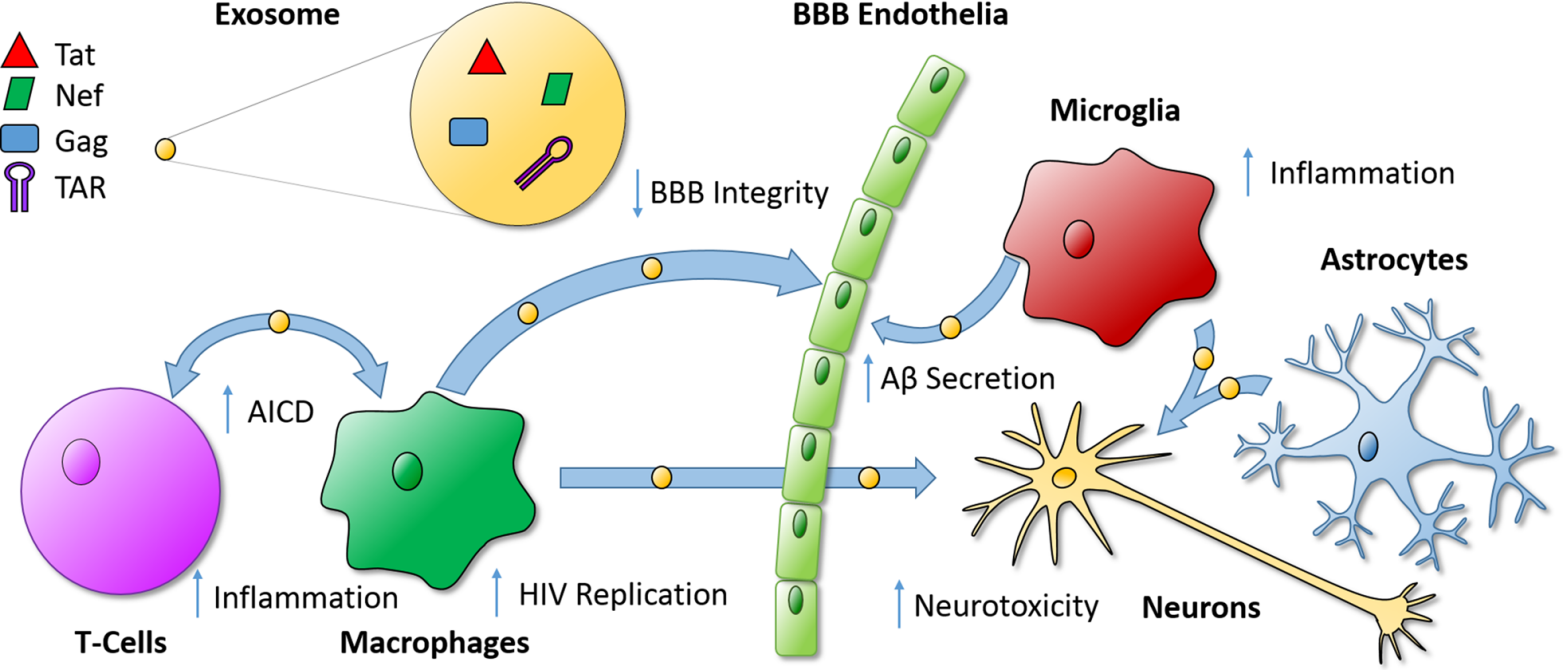
Mechanisms of exosome-mediated HIV pathogenesis. In vitro and ex vivo research has uncovered multiple mechanisms by which exosomal transport of viral proteins and RNA can cause deleterious effects in both infected and uninfected recipient cells. *Aβ* amyloid beta, *AICD* activation-induced cell death, *BBB* blood–brain barrier

Taken together, recent reports strongly indicate that exosomes bearing viral components act as mediators for HIV pathogenesis. Delivery of viral proteins in particular, irrespective of viral replication, induces inflammation, weakened immunity, and neurodegeneration that contributes to HAND (Table 1 and Fig. 1). Due to the failure of current ART options to counteract these effects, novel therapeutic approaches are a necessity for improving health and quality of life for people living with HIV.

## Therapeutic options to combat exosome-mediated HIV pathogenesis

**Fig. 2:**
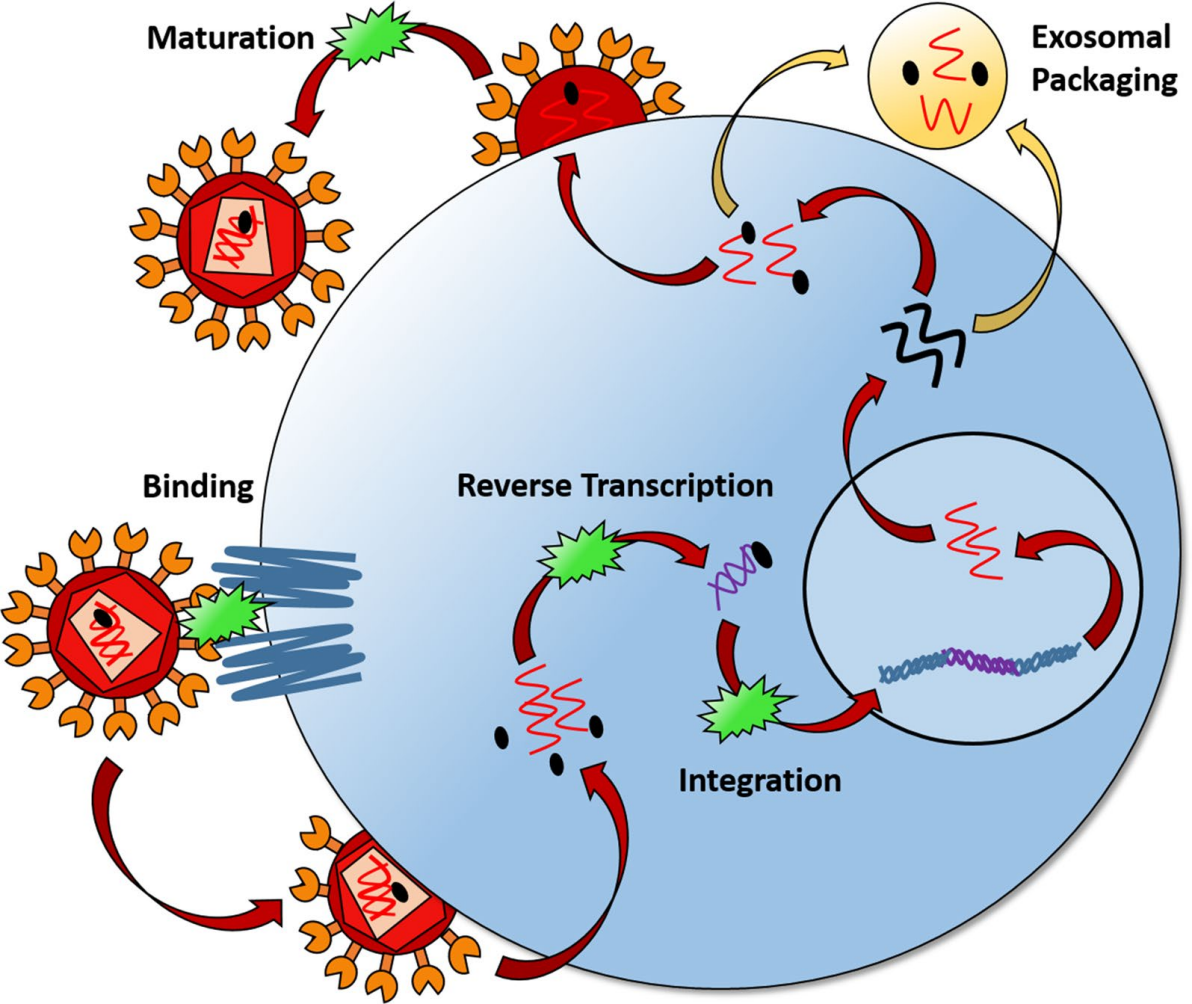
Packaging of HIV elements within exosomes despite ART. Modern ART combats multiple stages of the retroviral life cycle, however no current antiretroviral drug blocks the expression of HIV proteins and RNA from integrated viral DNA, or their subsequent sorting into exosomes and secretion from infected cells

A brief review of the mechanisms of action of the mainline ART drugs used to combat HIV is sufficient to illustrate why those medications fail to prevent exosomal packaging and secretion of retroviral elements [49, 120]. While entry, reverse transcriptase, and integrase inhibitors prevent the various stages prior to integration of the viral genome (thus averting complete infection of new cells) and protease inhibitors prevent the maturation of assembled virions, a gap exists between those two phases of the viral life cycle. The viral genomic DNA that is integrated into cells prior to ART administration can be transcribed, processed, translated, and secreted, despite the lack of productive virion formation under ART (Fig. 2). As such, current treatment regimens are inadequate to combat exosome-mediated HIV pathogenesis. Novel approaches are necessary to interfere with the processes that occur between retroviral genome integration and exosomal secretion of toxic viral elements.

There are several approaches that can be taken which ought to prevent exosome-mediated viral toxicity. The first and perhaps most direct approach is to interfere directly with exosome secretion. By disrupting the MVB biogenesis pathway, exosome secretion can be nearly abolished, which necessarily would prevent exosomal transport of viral proteins and RNA. The experimental drug GW4869 is an inhibitor of neutral sphingomyelinase, a lipid-metabolizing enzyme that has been found to be essential for proper MVB and exosome formation. It has been found to potently inhibit exosome production [121], and thus may be a candidate drug for disrupting a diverse array of exosome-mediated pathologies. Indeed, reports by Dinkins et al. [122] and Essandoh et al. [123] have shown that GW4869-induced suppression of exosome generation has beneficial effects in murine models of Alzheimer’s disease and sepsis, respectively. However, as Gould et al. noted in their original proposal of the Trojan Exosome Hypothesis, inhibition of exosome secretion and transmission may have significantly harmful side effects. Given the evidence that exosomes are critical vehicles for intercellular signaling [124], the nonspecific disruption of their generation could interfere with healthy tissue homeostasis. As such, global exosome suppression is not likely to be the optimal method of intervention.

A more narrowly targeted approach would be to interfere directly with the exosomal packaging of viral elements. Preventing the viral agents from being sorted into exosomes and secreted would avert their downstream effects without hampering normal intercellular communication. Doing so however requires detailed knowledge of the mechanisms of HIV packaging, which, as discussed previously, is a field in its infancy. However, some discoveries have been made which may have potential applications in this regard. In their 2012 study in which they uncovered the binding interactions between the “secretion modification region” of HIV Nef protein and the human cellular protein mortalin, Shelton et al. [94] synthesized a small peptide containing this same sequence.

That peptide, through competitive binding with mortalin, was able to block exosomal packaging of Nef without causing toxicity in transfected T-cells in vitro. Given even more recent research by the same laboratory showing that fusion peptides containing the Nef sequence may have applications in blocking exosome-mediated cancer metastasis [125], it seems likely that it may have significant utility as an inhibitor of multiple exosome-mediated maladies beyond HIV.

Another mechanism of viral protein sorting into exosomes that could be blocked is the interaction between Gag and the tetraspanin protein CD81. As discussed previously, both the exosomal secretion of Gag and HIV localization and assembly at the plasma membrane may take place at least in part through interactions with CD81 at TEMs. If that is the case, it follows that disruption of binding between Gag and CD81 would blockade both processes. There is some evidence for this hypothesis: Grigorov et al. [64] have shown that treatment of HIV-infected T-cells with antibodies against CD81 reduces viral release in vitro, presumably by preventing direct interaction between Gag and CD81, though this mechanism has not been fully elucidated. Interestingly, HCV also uses CD81 to propagate, specifically as an entry receptor [126]. Antibodies against CD81 have been used in an in vivo murine model of HCV infection, in which they were well-tolerated and showed potent antiviral activity as both a prophylactic and a means to prevent viral spread [127]. Much like the Nef-derived peptide inhibitor of mortalin, a number of peptides derived from HCV glycoproteins or from extracellular portions of CD81 have been developed to block HCV entry by competitive inhibition [128, 129]. An HCV-derived CD81 inhibitor could also have applications for interfering with the tetraspanin’s interaction with HIV Gag and subsequent loading of the viral protein into exosomes, though this is merely speculative.

Preventing the loading of HIV elements into exosomes is appealing, but it leaves open the possibility that viral proteins may still exert damaging effects on host tissues through direct secretion. To prevent such circumstances, the translation of HIV mRNA must be suppressed. In the past decade, a number of methods have been developed in the pursuit of such a goal. RNA interference, i.e. use of synthetic RNA to interfere with the translation, binding, or other activities of target RNAs, has shown potential as an antiretroviral therapeutic in vitro, and has been tested in clinical trials [130]. However, concerns over efficient delivery, and over the potential risks of viral mutation to escape RNA interference therapy, have hampered efforts to produce effective RNA-based antiretrovirals [131, 132]. Nevertheless, RNA interference therapy may yet prove useful in the future, if not as a standalone ART option then as a supplemental therapy given in combination with more traditional ART drugs to suppress the translation and export of HIV proteins in exosomes.

The transcription of integrated retroviral DNA is arguably an even more desirable stage of the HIV life cycle to target for preventing exosome-mediated toxicities. Preventing transcription, rather than translation, would suppress the expression of both viral proteins and HIV-derived miRNA, which are also packaged and secreted into exosomes [44, 48]. While there are some cellular factors that can promote transcription of the HIV genome, the viral Tat protein is the most appealing target for inhibition of transcription, as its interaction with TAR RNA is essential for significant expression of viral genes, and because it has no close cellular homologs [133]. Some of the first Tat inhibitors were developed in the 1990s, and were simply circularized TAR RNA decoys that could compete with genomic TAR to bind Tat and inhibit viral transcription [134]. Tat-mimetic peptides that bind TAR have also been investigated, with some having potential efficacy in inhibiting reverse transcription as well [73]. More current research into inhibition of retroviral transcription has focused on small molecule Tat inhibitors, both in terms of discovery of novel inhibitors and repurposing of existing drugs [135]. For an example of the latter, Hayashi et al. [136] recently reported that levosimendan, an FDA-approved drug used in the treatment of heart failure, effectively blocked interactions between Tat and the HIV genomic 5′ long terminal repeat, indicating its potential use in ART as a transcription inhibitor with an already-established safety record in patients. Another group has developed a novel inhibitor of Tat, didehydro-cortistatin A (dCA), which interferes with viral transcription and elongation at Tat’s TAR-binding site [135]. In a recent publication, the same group presented evidence that, in addition to direct inhibition of Tat-mediated transcription, dCA is also able to silence further viral expression through epigenetic modifications that restrict the HIV promoter [137]. The authors go so far as to propose that this epigenetic silencing may present a “functional cure” for HIV by permanently blocking viral transcription in infected patients, though of course much more research would be necessary to substantiate those claims. Regardless, the established direct anti-transcriptional effects of dCA or another Tat-TAR inhibitor could be sufficient to block the expression and loading of both HIV proteins and RNA molecules into exosomes.

There may also be endogenous factors with HIV-suppressive effects that could be exploited to restrict retroviral transcription. For example, CD8+ T-cells are known to secrete an antiretroviral factor in vitro and ex vivo that safely and potently suppresses binding of the RNA polymerase II complex with the viral genome [138]. In 2009, Tumne et al. [139] reported that this factor may be membrane-bound and secreted via exosomes, which could make it a viable candidate as a vesicle-based therapeutic. Similarly, research from the Okeoma laboratory has shown that exosomes collected from human semen have antiretroviral activity, suppressing viral RNA levels via disruption of interactions between the HIV genome and both host and viral transcription factors (e.g. NF-κB and Tat) [140–142]. Once the factors involved in these interactions are identified, and their exact mechanisms of antiretroviral activity fully elucidated, they may yield novel therapeutic targets for suppressing HIV transcription and subsequent exosome-mediated pathogenesis, though much further research is required on that front.

Each approach to treating exosome-mediated HIV pathogenesis has pros and cons. Global suppression of exosome secretion is likely excessively broad in its effects, while specific blocking of known mechanisms of viral exosome loading may be too narrow. RNA interference therapy would block the expression of HIV proteins, but could prove ineffective due to mutation of the viral genome. That leaves blockade of transcription of the viral genome with perhaps the greatest potential for novel therapeutics. Transcription inhibitors, by blocking the expression of any viral elements, should in principle suppress any exosome-mediated viral toxicities, and would serve as a novel class of ART drugs as well, closing the previously described gap in current treatment paradigms.

## Conclusion

It has become evident that HIV is deeply integrated with the pathways of exosome biogenesis. It assembles at the same membrane regions through interactions with tetraspanins and lipid rafts. Its major pathogenic elements interact directly with the host protein and RNA sorting machinery by multiple apparently distinct mechanisms. HIV elements are not only sorted into exosomes, but can facilitate loading of host proteins as well, to pathogenic ends. While discussion continues regarding the origin of these interactions (by divergence from a common vesicular pathway or through selection for retroviral “hijacking” of established cellular phenomena), the consequences are quickly becoming apparent. Exosomal transport of HIV proteins and RNA could potentially contribute to chronic inflammation, leakiness of gut or BBB endothelia, and long-term neurological dysfunction. It may also contribute to other HIV-associated organ and tissue damage mediated by viral elements, such as HIV-associated nephropathy [143]. The full scope of viral pathogenesis by means of exosomal transport has yet to be fully explored.

Current ART regimens cannot protect infected individuals from exosome-associated viral toxicities. Modern antiretrovirals are designed to prevent either the formation of mature virions or the infection of new cells. This tactic is sufficient to suppress viral load and restore CD4 T-cell counts to healthy levels, but does not address the root cause of exosome-mediated pathogenesis: transcription and translation of the viral genome. Put simply, as long as latently infected cells are capable of producing functional viral RNA and proteins, exosome-associated HIV toxicities will persist under current ART. This fact underscores the need for novel treatment stratagems to combat this portion of the HIV life cycle. The ideal solution would be the discovery of the elusive functional cure for HIV infection, be it by “shock and kill” [144] or “block and lock” [137] approaches, by targeted gene editing to eliminate functional proviral DNA [145]. However, until such time as a safe and effective cure is found, alternative antiretroviral drugs are needed. HIV transcription inhibitors would eliminate the source of the problem, but any intervention that is able to abrogate the exosomal packaging and transmission of viral elements is likely to have a robust therapeutic effect and to have a long-term positive impact on the health and quality of life of people living with HIV.

## Abbreviations

AICD: activation-induced cell death
ART: antiretroviral therapy
BBB: blood–brain barrier
dCA: didehydro-cortistatin A
ESCRT: endosomal sorting complex required for transport
HAND: HIV-associated neurocognitive disorders
HCV: hepatitis C virus
HIV: human immunodeficiency virus
MDM: monocyte-derived macrophage
MVB: multivesicular body
TAR: trans-activation response element
TEM: tetraspanin-enriched microdomain
